# Applying a Sensing-Enabled System for Ensuring Safe Anterior Cingulate Deep Brain Stimulation for Pain

**DOI:** 10.3390/brainsci9070150

**Published:** 2019-06-26

**Authors:** Yongzhi Huang, Binith Cheeran, Alexander L. Green, Timothy J. Denison, Tipu Z. Aziz

**Affiliations:** 1 Oxford Functional Neurosurgery Group, Nuffield Departments of Surgical Sciences, University of Oxford, Oxford OX3 9DU, UK; 2 Institute of Biomedical Engineering, University of Oxford, Oxford OX3 7DQ, UK

**Keywords:** deep brain stimulation, anterior cingulate cortex, seizures, after-discharge, local field potential, chronic pain

## Abstract

Deep brain stimulation (DBS) of the anterior cingulate cortex (ACC) was offered to chronic pain patients who had exhausted medical and surgical options. However, several patients developed recurrent seizures. This work was conducted to assess the effect of ACC stimulation on the brain activity and to guide safe DBS programming. A sensing-enabled neurostimulator (Activa PC + S) allowing wireless recording through the stimulating electrodes was chronically implanted in three patients. Stimulation patterns with different amplitude levels and variable ramping rates were tested to investigate whether these patterns could provide pain relief without triggering after-discharges (ADs) within local field potentials (LFPs) recorded in the ACC. In the absence of ramping, AD activity was detected following stimulation at amplitude levels below those used in chronic therapy. Adjustment of stimulus cycling patterns, by slowly ramping on/off (8-s ramp duration), was able to prevent ADs at higher amplitude levels while maintaining effective pain relief. The absence of AD activity confirmed from the implant was correlated with the absence of clinical seizures. We propose that AD activity in the ACC could be a biomarker for the likelihood of seizures in these patients, and the application of sensing-enabled techniques has the potential to advance safer brain stimulation therapies, especially in novel targets.

## 1. Introduction

Deep brain stimulation (DBS) is an established therapy for movement disorders. It has several advantages over lesioning techniques. For example, therapy-induced side effects are considered to be reversible, and DBS can be ‘dosed’ as symptoms evolve [1]. Interestingly, the first DBS surgeries were performed for chronic post-stroke pain [2]. Cingulotomy has historically been used to target the affective component of pain, for example intractable pain associated with terminal cancer [3,4,5]. However, side effects are common, such as impairments of attention and cognition [6,7]. As an alternative to destructive lesioning, anterior cingulate cortex (ACC) DBS implants were offered to patients with severe, medically-refractory pain, where established targets, such as sensory thalamus and periventricular/periaqueductal grey, had failed or where pain was too poorly localized to consider these targets [8,9].

The success of ACC stimulation in patients deemed refractory to other medical and surgical interventions was tempered by the onset of recurrent stereotyped neurological events after 12–60 months of active stimulation in approximately 18% of the patients [10]. Some patients experienced recurrent seizures, and stimulation had to be markedly reduced or switched off completely as a conservative solution to ameliorate side effects; at these settings, effective pain relief was lost [10]. However, how ACC stimulation affects brain function and induces seizures remains unclear.

Studies have suggested several neurophysiological biomarkers potentially associated with seizures, e.g., stimulation-elicited after-discharges (ADs) [11,12]. Cortical stimulation can induce ADs, sometimes followed by clinical seizures, whether or not those regions are known to cause spontaneous seizures [11,13,14]. Stimulation parameters for inducing ADs have shown considerable within- and between-subject variability, but in general ADs can be elicited with sufficient stimulus intensity and duration [15,16,17].

With the advent of recent implant technologies, the effects of DBS on brain neural activities can be chronically investigated by measurements of local field potentials (LFPs) in the brain. Prior animal studies have demonstrated the ability to detect AD activity in LFPs using implanted DBS leads in various brain regions [18,19]. Here, we applied this technology to investigate the effect of ACC stimulation on brain activity in patients with chronic pain and aimed to elucidate safe stimulation parameters that maintained adequate pain relief without inducing seizures.

## 2. Materials and Methods

### 2.1. Subjects

Three patients with chronic pain who experienced recurrent seizures during the course of DBS therapy were investigated. All patients initially underwent bilateral implantation of DBS electrodes (Model 3387, Medtronic^®^, Minneapolis, MN, USA) into the ACC at The John Radcliffe Hospital, Oxford, UK. The surgical procedure has been previously described [8]. Details of the patients are reported in Table 1. None of the patients had suffered seizures prior to their initial surgery. Pre-operative MRI and post-operative CT scans did not reveal any relevant structural abnormalities or complications, such as hemorrhage or ischemia.

**Figure 1:**
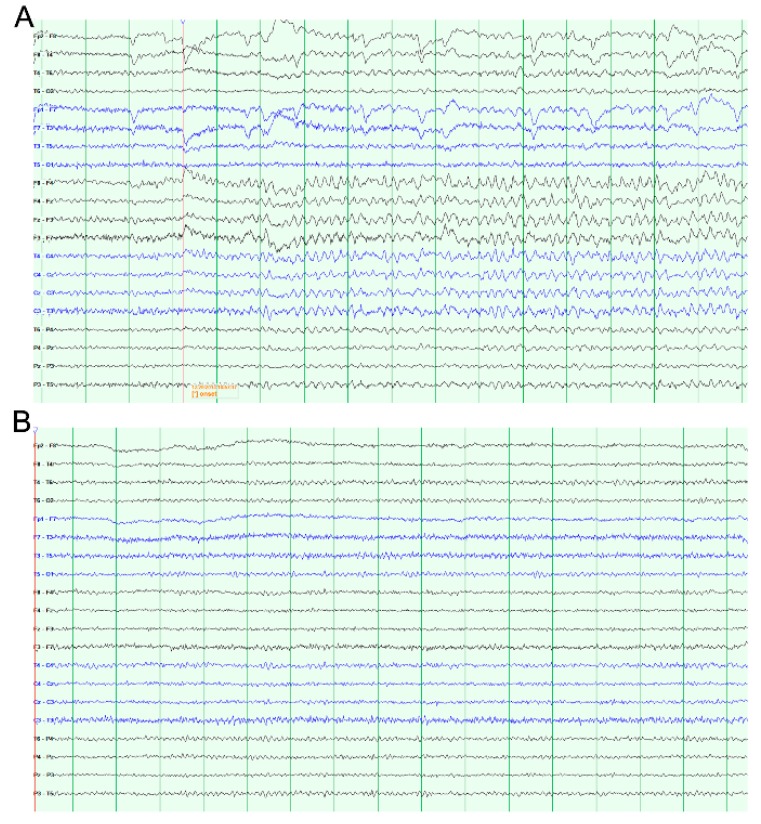
Example EEG recordings from patient 1. (**A**) Ictal EEG showing rhythmical, symmetrical 6 Hz theta slow wave activity across the frontocentral regions. (**B**) EEG showing normal background activity.

However, stereotyped neurological events, clinically diagnosed as seizures, were reported in these patients after some period of effective therapy. Medical management with multiple anti-epileptics was only transiently effective (weeks to months) before seizures recurred. Patient 2 did not take anti-epileptic drugs as he preferred altering the stimulation settings rather than medication. Video-electroencephalograph telemetry (vEEG) was used to investigate and detect seizures, initially in the first case (Figure 1). Reducing stimulation amplitude to levels below the threshold for seizure induction, based on multiple vEEG tests and clinical review, eliminated both the clinical seizures and the benefit of pain relief. Despite the risk of seizure induction, the patients requested reinstatement of stimulation to re-capture pain relief.

### 2.2. Stimulation and Local Field Potential Recordings

To further investigate the relationship between the stimulation and clinical events, a sensing-enabled neurostimulation system (Activa PC + S, Medtronic^®^) was implanted chronically, under the Medicines and Healthcare Products Regulatory Agency (MHRA) humanitarian exemption approval. This system allows for concurrent stimulation and recording from implanted DBS leads, and was used in a prior study to measure AD activity in an animal model, exploring network behavior in epilepsy [19].

Stimulation titration tests were systematically performed in patient 1 to investigate the effect of stimulation intensity on ACC neural activity. In particular, we explored whether ADs could be induced by ACC stimulation. Unilateral stimulation was increased from 0 V to target voltage (from 1 V to 6 V, 1 V steps) and then immediately switched off, with a stimulation-off interval of several seconds between steps. Ipsilateral and contralateral LFPs were recorded simultaneously during the same period. In addition, bilateral stimulation was also tested. The implantable pulse generator (IPG) was replaced with a second Activa PC + S system 13 months after the first implant of the Activa PC + S system due to depleted battery. Thereafter, bilateral stimulation at therapeutic amplitudes, using cycled stimulation with ramping, was explored to re-capture pain relief whilst minimizing seizures. Cycled stimulation on/off durations were selected based on results from the stimulation testing trials indicating that pain relief could be achieved with 3 min of stimulation, using therapeutic amplitudes, but could be lost 11 min later in this patient (Table 1). LFPs in the ACC were measured to investigate whether these stimulation patterns would induce ADs. Additional LFP recordings were collected during periods of chronic stimulation at home using the embedded loop recorder in the device [20,21].

The know-how learned in patient 1 was applied to patients 2 and 3. As unilateral ACC stimulation was found to be ineffective, bilateral stimulation without ramp, and with slowly ramped on/off, was tested, and LFPs were measured simultaneously. Subsequently, the stimulation pattern using cycling with ramp, shown through LFP measurements to avoid ADs, was applied for chronic treatment. Unfortunately, a month after IPG implant surgery, the sensing-enabled stimulation system had to be removed because of an infection in patient 3. During this period, the stimulation was clinically effective for pain relief without inducing seizures.

In all tests, electrical stimulation was delivered using a bipolar configuration between electrode contacts 0 (the deepest contact) and 3. Stimulation frequency and pulse width (typical therapy parameters 130 Hz, 450 μs) were fixed. All LFPs were recorded in a bipolar mode using the middle two contacts (1–2) of the electrodes (0.5 Hz pre-amplifier high-pass filtering, 100 Hz pre-amplifier low-pass filtering, 422 Hz sampling rate).

### 2.3. Data Analysis

LFP data were analyzed using custom scripts written in MATLAB (Version 9.1, MathWorks, Natick, MA, USA). To characterize the dynamic changes of neural activity, time-frequency representations of LFPs were performed using the short-time Fourier transform with a Hanning time window of 0.5 s and overlap of 0.45 s. These parameters provided a time resolution of 0.5 s and a frequency resolution of 2 Hz. Stimulation onset was identified as a period where observable high amplitude artifacts in the raw LFPs were accompanied by obvious 130 Hz stimulation frequency in the spectrograms. An AD episode was defined as the state with a sustained high amplitude, seizure-like activities in the raw LFPs and confirmed through elevated power across multiple frequency bands in the spectrograms.

**Figure 2:**
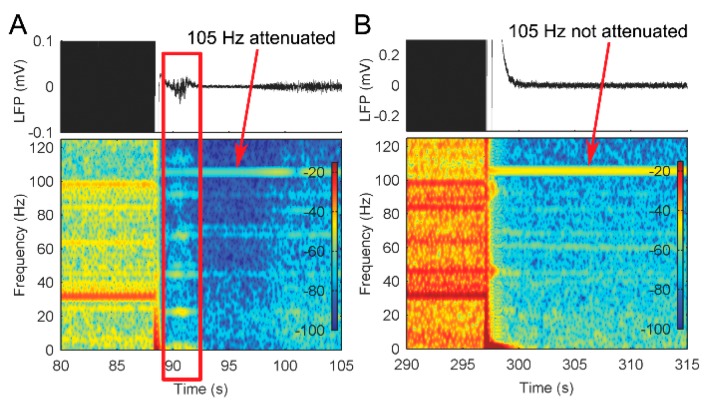
The use of a test tone to monitor the integrity of the bioelectric amplifier in measuring local field potentials (LFPs). (**A**) LFP measurement using an amplifier with a gain of 2000. The line box illustrates an example of distortion of signals induced by the amplifier recovering. (**B**) LFP measurement using an amplifier with a gain of 250.

Sensing channel saturation with large stimulation was a concern. To ensure a robust LFP measurement, a continuous monitoring approach was used to determine the reliability of the received signals [22]. Briefly, a continuous test tone at a discrete frequency (105 Hz) outside of the physiological band of interest was injected into the signal chain during recording through a parallel channel. If this tone’s amplitude was compromised due to amplifier saturation, alternative signal chain parameters would be chosen, such as reducing the amplifier gain. For example, in our study, if the test tone shows amplifier saturation then the signal artifact following stimulation can look like a seizure activity (Figure 2A). In such cases, the amplifiers’ gain was reduced to ensure the recording of reliable LFPs (Figure 2B).

## 3. Results

### 3.1. AD Activity in ACC LFPs Is Induced Following Stimulation

**Figure 3:**
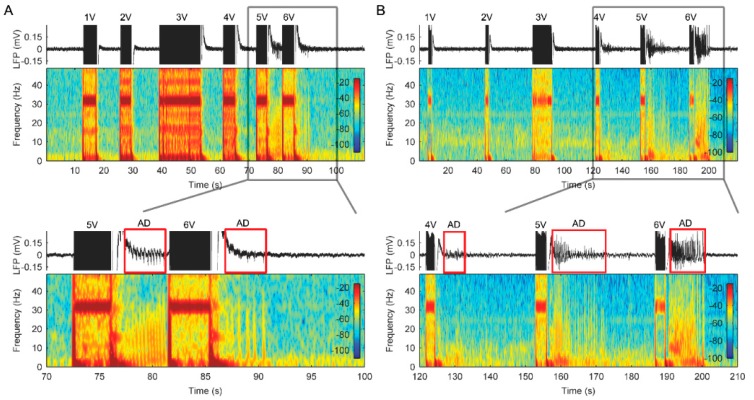
Effects of different unilateral stimulation amplitudes on the LFP recordings in the anterior cingulate cortex (ACC) from patient 1. (**A**) Bipolar LFP recordings and corresponding spectrograms from the left ACC during unilateral stimulation with increasing amplitudes. (**B**) Bipolar LFP recordings and corresponding spectrograms from the right ACC during unilateral stimulation with increasing amplitudes. Bottom panels show greater details illustrating several examples of after-discharges that occurred following cessation of stimulation.

**Figure 4:**
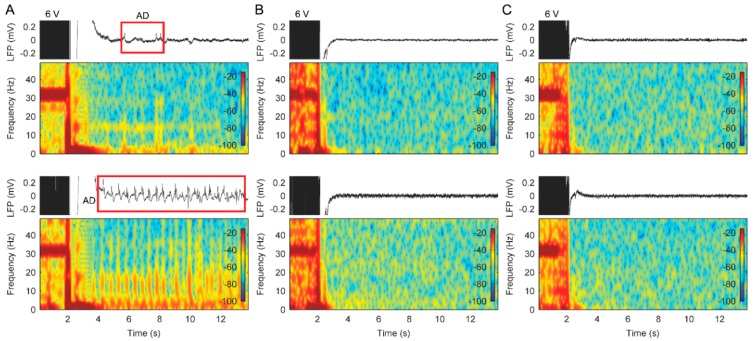
Effects of bilateral stimulation with/without a stimulus ramp on the LFP recordings in the bilateral ACC from patient 1. (**A**) Raw LFP recordings and corresponding spectrograms in the left (top panels) and right ACC (bottom panels) during stimulation without a stimulus ramp. (**B**) Raw LFP recordings and corresponding spectrograms in the left (top panels) and right ACC (bottom panels) during stimulation with a 4 s stimulus ramp. (**C**) Raw LFP recordings and corresponding spectrograms in the left (top panels) and right ACC (bottom panels) during stimulation with an 8 s stimulus ramp.

Stimulation titration tests revealed that the characteristics of LFP changes in the ACC were dependent upon the stimulation amplitudes. Figure 3 provides an illustration of the effects of unilateral stimulation amplitudes on ACC LFPs from patient 1. The ADs were induced following a stimulation at a threshold of 5 V on the left lead and 4 V on the right lead, respectively (Figure 3). Bilateral stimulation with 6 V amplitude on both leads also induced significant ADs in right ACC and slight ADs in left ACC (Figure 4A). The ADs in the ACC could also be observed when we repeated the titration tests after 1 month of bilateral therapeutic stimulation at 3.5 V (not shown). The AD threshold to stimulation was determined based on these tests. During the periods of measuring these LFPs, no clinical seizures were reported. Based on these LFP measurements, bilateral stimulation therapy with amplitudes below the AD threshold level was applied to attempt to prevent seizures and obtain pain relief. However, pain relief was inadequate, although no seizures were reported for approximately 12 months.

### 3.2. Stimulation with Slowly Ramped on/off during Cycling Successfully Eliminates ADs

Subsequently, based on the rationale detailed in the discussion, we tested a programming feature that allowed stimulation to be slowly ramped on/off during the cycling, rather than stimulation being started or stopped abruptly. When stimulation was delivered, using a pattern consisting of cycled stimulation, stimulation ramped down from the maximum amplitude to 0 V in 8 s and a high amplitude (6 V) that previously resulted in ADs, we did not observe the typical AD activities following the stimulation in patient 1 (Figure 4C). With these stimulation patterns for therapy, good pain relief was again reported, and the patient was discharged with the device programmed to collect additional LFP recordings over time.

**Figure 5:**
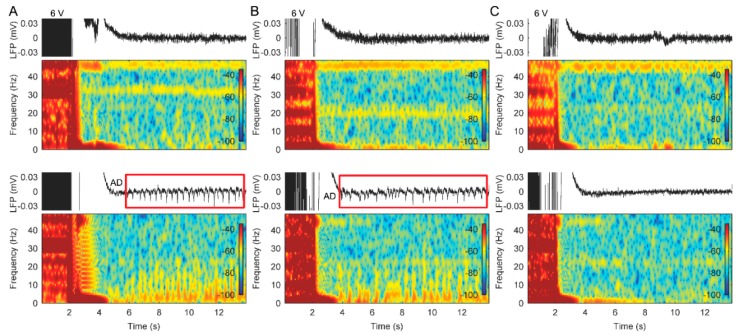
Effects of bilateral stimulation with/without a stimulus ramp on the LFP recordings in the bilateral ACC from patient 2. (**A**) Raw LFP recordings and corresponding spectrograms in the left (top panels) and right ACC (bottom panels) during stimulation without a stimulus ramp. (**B**) Raw LFP recordings and corresponding spectrograms in the left (top panels) and right ACC (bottom panels) during stimulation with a 4 s stimulus ramp. (**C**) Raw LFP recordings and corresponding spectrograms in the left (top panels) and right ACC (bottom panels) during stimulation with an 8 s stimulus ramp.

LFPs recordings from patient 2 also showed that ADs were observed in the right ACC when using cycled stimulation patterns without a stimulus ramp and with a 4 s ramp; however, the AD activity disappeared when using stimulation with an 8 s ramp (Figure 5). The patient gained pain relief and no seizures during the test, using the cycled stimulation pattern at 6 V amplitude with an 8 s ramp. Therefore, we applied this stimulation pattern for chronic therapy in the patient.

**Figure 6:**
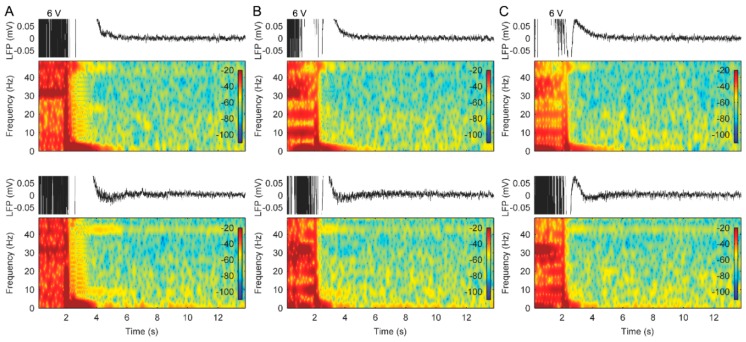
Effects of bilateral stimulation with/without a stimulus ramp on the LFP recordings in the bilateral ACC from patient 3. (**A**) Raw LFP recordings and corresponding spectrograms in the left (top panels) and right ACC (bottom panels) during stimulation without a stimulus ramp. (**B**) Raw LFP recordings and corresponding spectrograms in the left (top panels) and right ACC (bottom panels) during stimulation with a 4 s stimulus ramp. (**C**) Raw LFP recordings and corresponding spectrograms in the left (top panels) and right ACC (bottom panels) during stimulation with an 8 s stimulus ramp. Note that the after-effects observed during 3–6 s were confirmed to be due to sensing channel recovery using the test-tone method.

Although the system in patient 3 was removed due to infection at one month, the limited LFP recordings also revealed that there were no ADs using cycled stimulation with a ramp. Interestingly, in this case, we also did not observe the ADs when using stimulation without a ramp (Figure 6). Before system removal, the patient achieved pain relief and was seizure free.

### 3.3. The Use of Cycled Stimulation with Slow Ramps Provides Sustained Pain Relief without Seizures

**Figure 7:**
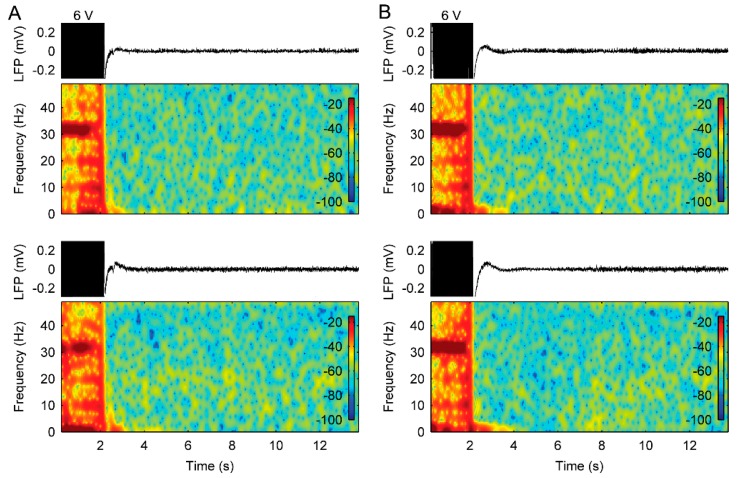
Long-term effects of bilateral therapeutic stimulation with an 8 s ramp on the LFP recordings in the bilateral ACC from patient 1. (**A**) Raw LFP recordings and corresponding spectrograms in the left (top panels) and right ACC (bottom panels) during stimulation for 5 months. (**B**) Raw LFP recordings and corresponding spectrograms in the left (top panels) and right ACC (bottom panels) during stimulation for 9 months.

At the follow up, the parameter settings of stimulation that provided pain relief without ADs resulted in sustained therapeutic benefit without side-effects. The long-term LFP recordings, obtained during periods of chronic stimulation at home in patient 1, also showed no indication of ADs being triggered by chronic-cycled stimulation with a ramp (Figure 7). At the last follow-up, patient 1 had been seizure free (self-reported) for 17 months and patient 2 had been seizure free (self-reported) for 6 months.

## 4. Discussion

Neuromodulation can be an effective approach to pain management in patients that have exhausted medical therapies. However, the risk of adverse events with cortical stimulation, as reported here, needs to be addressed. For example, epidural motor cortex stimulation and repetitive transcranial magnetic stimulation have been explored for a variety of pain syndromes with variable success, but the induction of seizures has been reported as one of the more serious adverse events [23,24,25]. Stimulation induced ADs are common during cortical mapping for epilepsy surgery, yet, despite decades of clinical observation, the cellular and network mechanisms underlying their generation remain areas of active investigation [26]. However, it is generally agreed that a disruption in excitatory-inhibitory balance results in the hyperexcitable state associated with these phenomena. During stimulation, the inhibitory drive on the post-synaptic neurons is increased, resulting in hyperpolarization. However, upon termination of the stimulus train, a phenomenon known as “post-inhibitory rebound excitation” can occur [27]. This rebound depolarization leads to a strong excitatory discharge in the primary neurons and may be one of the cellular mechanisms responsible for the generation of ADs. Using the same implantable device described here, this pattern of inhibition during stimulation, followed by strong excitatory bursts upon stimulus termination, has been observed in LFPs, chronically recorded from the sheep hippocampus [19].

This study indicates that AD activity in the ACC could be a biomarker for the likelihood of seizures. The relationship between the generation of ADs and the initial appearance of clinical seizures in these patients is unclear. Their recurrent seizures developed during a period where DBS was delivered in a continuous (not cycled) manner and could more likely be related to a kindling-like phenomenon. In animal models, classical kindling typically involves the application of periodic subthreshold stimuli to evoke network synchronization, which gradually induces long-lasting neuronal changes that eventually lead to spontaneous seizures. However, other kindling models employ higher level, more continuous stimulation, above the AD threshold, and result in a more rapid induction of epileptogenesis [28].

Stimulation with the cycle mode, rather than the prolonged continuous stimulation, has been proposed to reduce the risk of seizures [29]. Moreover, in an attempt to minimize the likelihood of ADs, a stimulation cycle that was slowly ramped off, rather than stopped abruptly, was evaluated in these patients. When stimulation was delivered using this pattern, the typical post-stimulation burst of spiking activity was not observed, even at intensity levels above those that earlier produced ADs, using the sensing capability of the implanted brain-machine-interface. Due to the large stimulus artifact, it was not possible to conclusively determine whether any spiking/ictal activity was present during stimulation. However, the stimulation pattern with a ramp appeared to avoid the generation of ADs following stimulation, possibly due to a reduction in the post-inhibitory rebound. Importantly, it has allowed for two patients to achieve long-term seizure freedom and pain relief.

Future systems would benefit from continuous monitoring of neural activity. Our data suggests that the likelihood of AD occurrence can fluctuate depending on the functional state of the stimulated network at that time [16]. This may explain why the LFPs recorded from patient 3 did not show ADs when using a stimulus without a ramp, which also suggests the importance of long-term monitoring and adaptive algorithms. Implanted sensing-enabled interfaces have the capability to chronically monitor for AD/ictal types of activity, based on spectral characteristics, and options to reduce or turn off stimulation, if detected [21,30]. Moreover, the sensing-enabled interfaces could be easily automated and run in the background and allow for more automated processing in the future. This type of closed-loop approach may potentially minimize or prevent stimulation-induced adverse events, such as those observed in this study.

New applications of DBS of new targets for therapy delivery continue to be explored; however, in most cases, the default stimulation parameters selected are based upon those that have been effective in the currently approved movement disorder therapies [31]. When exploring new therapies, a commonly accepted concept in neuromodulation is that side-effects and adverse events can be eliminated by simply turning off the device. However, the patients then fail to obtain symptomatic relief, and, as illustrated in this case series, there is a potential for sustained adverse events, even in the absence of stimulation. This caveat is important, as several recent large trials of DBS for new indications have not yielded positive outcomes [32,33,34] and a post-hoc review questioned whether stimulation parameters were adequately dosed. The unique opportunity to directly observe stimulation effects on the implanted structure [35,36] or neural network [37,38,39] targeted for therapy provided by sensing-enabled systems may usher in a new era, where DBS programming is informed by objective electrophysiological measures in conjunction with clinical observations, hopefully leading to safer and more effective therapies.

## 5. Conclusions

The events of unforeseen consequences following ACC DBS serve as a clarion call to those working in the field of neuromodulation. This report revealed that use of sensing-enabled systems could help to understand relationship between ACC stimulation and side-effects (seizures in these series), suggesting sensing-enabled techniques have the potential to advance safer brain stimulation therapies, especially in novel targets.

## Figures and Tables

**Table 1:** Demographics, etiologies, stimulation parameters, and clinical events of patients.

Patient	Age at Surgery/Sex	Etiology	Onset of Seizure after Surgery	Seizure Symptoms	DBS Settings at Onset of Seizures	Anti-Epileptic Drugs	DBS Settings with Seizure Free	Follow-Up
1	46/F	Whole spine pain secondary to multiple spinal interventions	20 months	1) Focal non-motor onset with impaired awareness 2) Nocturnal generalized tonic-clonic seizures (maximum frequency reported: 1 event per month, lasting up to 45 min)	5 V 130 Hz 450 μs	Levetiracetam Clobazam Sodium valproate	6 V 130 Hz 450 μs 8-s ramp 3 min ON/11 min OFF	Seizure free for 17 months
2	51/M	Whole body pain secondary to excision of ependymoma of cervical spinal cord	60 months	Focal non-motor onset with impaired awareness (maximum frequency reported: 1 event per h)	8.5 V 130 Hz 450 μs	no	6 V 130 Hz 450 μs 8-s ramp1 min ON/1 min OFF	Seizure free for 6 months
3	49/M	Right hemi body pain secondary to posterior fossa decompression for Arnold–Chiari malformation	12 months	Focal non-motor onset with impaired awareness (maximum frequency reported: 50 events per day)	8.5 V 130 Hz 450 μs	Levetiracetam Oxcarbazepine	6 V 130 Hz 450 μs 8-s ramp1 min ON/1 min OFF	Seizure free for 1 month (then system removed due to infection)
